# SNPs in cytochrome P450 genes decide on the fate of individuals with genetic predisposition to Parkinson’s disease

**DOI:** 10.3389/fphar.2023.1244516

**Published:** 2023-08-04

**Authors:** Polina Petkova-Kirova, Stephan Baas, Gudrun Wagenpfeil, Philip Hartz, Marcus Michael Unger, Rita Bernhardt

**Affiliations:** ^1^ Institut für Biochemie, Fachbereich Biologie, Naturwissenschaftlich-Technische Fakultät, Universität des Saarlandes, Saarbrücken, Germany; ^2^ SHG Kliniken, Saarbrücken, Germany; ^3^ Institut für Medizinische Biometrie, Epidemiologie und Medizinische Informatik, Universität des Saarlandes, Homburg, Germany; ^4^ Klinik für Neurologie, SHG Kliniken Sonnenberg, Saarbrücken, Germany

**Keywords:** Parkinson’s disease, cytochromes P450, POR, Adx, eicosanoids, cholesterol, vitamin D, retinoic acid

## Abstract

Parkinson’s disease (PD) is one of the most frequent neurological diseases affecting millions of people worldwide. While the majority of PD cases are of unknown origin (idiopathic), about 5%–10% are familial and linked to mutations in different known genes. However, there are also people with a genetic predisposition to PD who do not develop the disease. To elucidate factors leading to the manifestation of PD we compared the occurrence of single nucleotide polymorphisms (SNPs) in various cytochrome P450 (P450) genes in people with a genetic predisposition and suffering from PD (GPD) to that of people, who are genetically predisposed, but show no symptoms of the disease (GUN). We used the PPMI (Parkinson’s Progression Markers Initiative) database and the gene sequences of all 57 P450s as well as their three redox partners. Corresponding odds ratios (OR) and confidence intervals (CI) were calculated to assess the incidence of the various SNPs in the two groups of individuals and consequently their relation to PD. We identified for the first time SNPs that are significantly (up to 10fold!) over- or under-represented in GPD patients compared to GUN. SNPs with OR > 5 were found in 10 P450s being involved in eicosanoid, vitamin A and D metabolism as well as cholesterol degradation pointing to an important role of endogenous factors for the manifestation of PD clinical symptoms. Moreover, 12 P450s belonging to all P450 substrate classes as well as POR have SNPs that are significantly under-represented (OR < 0.2) in GPD compared to GUN, indicating a protective role of those SNPs and the corresponding P450s regarding disease advancement. To the best of our knowledge our data for the first time demonstrate an association between known PD predisposition genes and SNPs in other genes, shown here for different P450 genes and for their redox partner POR, which promote the manifestation of the disease in familial PD. Our results thus shed light onto the pathogenesis of PD, especially the switch from GUN to GPD and might further help to advance novel strategies for preventing the development or progression of the disease.

## Introduction

Parkinson’s disease (PD) is the second most common neurodegenerative disorder worldwide. According to the World Health Organization, disease prevalence has doubled in the last 25 years. For the year 2019, over 8.5 million people have been shown to suffer from PD with estimated 329,000 deaths caused by the disease, an increase of over 100% since 2000 (https://www.who.int/news-room/fact-sheets/detail/parkinson-disease). The reason for the degeneration of *Substantia nigra pars compacta* and the related loss of dopaminergic neurons, main characteristics of the disease, is not yet completely known. Therefore, treatment of PD remains symptomatic and new biomarkers for early diagnostics and novel approaches as well as candidates for causative and effective treatment are pursued.

Very recently we published a novel approach analyzing a possible association between the pathogenesis of PD and single nucleotide polymorphisms (SNPs) in cytochrome P450 (P450) genes (Hartz et al., 2022). The Parkinson’s Progression Markers Initiative (PPMI) database was used to evaluate possible relations between variations in the genes of the 57 human P450s and their 3 redox partners, a cytochrome P450 reductase (POR), adrenodoxin (Adx) and adrenodoxin reductase (AdR), and the origin and development of PD. Statistical analysis of the data was applied to calculate corresponding odds ratios (OR) and confidence intervals (CI) to estimate the link between the occurrence of a particular SNP in the genes of the 60 proteins and the incidence of PD. It was shown that SNPs in 26 out of 57 P450s and their three redox partners were significantly over-represented (with OR values >5) in patients with a genetic predisposition to PD (GPD) compared to healthy subjects (HC), taken as controls. Three main groups of P450s were shown to contain those SNPs: 1) xenobiotics-metabolizing P450s demonstrating the biggest accumulation of highly over-represented SNPs, posing a major role for toxic compounds in the pathogenesis of PD, 2) P450s involved in eicosanoid metabolism, confirming the relation of PD to inflammation and 3) P450s involved in the degradation of cholesterol (CYP46A1, CY7B1, CYP39A1), indicating a prominent role of brain cholesterol metabolism for the risk to develop PD. Additionally, POR, required for electron transfer from NADPH to microsomal P450s, displayed nine SNPs with OR > 5 in GPD and thus also seems to play an important role in the etiology of PD (Hartz et al., 2022). In contrast, in idiopathic PD patients (IPD), SNPs with OR values >5 were only described for CYP46A1 as well as Adx and POR. However, SNPs with OR values between 2 and 5 are much more abundant, especially in the group of xenobiotics-converting enzymes like CYP2E1 and CYP2C8 (Hartz et al., 2022).

While the majority of PD cases worldwide are idiopathic, about 10%–15% of all patients and about 25% of early-onset patients have a family history (first degree relatives) of PD and approximately 5%–10% are ascertained with a defined genetic predisposition (Sellbach et al., 2006; Lesage and Brice, 2009; Nalls et al., 2019). Mutations in seven genes have been unambiguously linked to typical familial PD: *SNCA*, *LRRK2*, *VPS35*, *PRKN*, *PINK1*, *GBA* and *DJ-1* (Bandres-Ciga et al., 2020), but most of the patients with a genetic predisposition studied so far show changes in the *LRRK2* (leucine rich repeat kinase 2) gene leading to changes in kinase activity and *a*-synuclein and mitochondrial impairments (Di Maio et al., 2018). Interestingly, in the PPMI database there are also individuals who have a genetic predisposition to PD, but do not show any signs of the disease (designated in our study as GUN). It is not clear at the moment which factors are necessary to promote the progression of the disease at the background of an existing genetic predisposition.

Thus, the aim of our present study was to analyze the differences in the occurrence of SNPs in the P450 genes and the genes of their three redox partners in GPD and GUN individuals to further shed light on factors eliciting disease manifestation.

## Materials and methods

Information for the genetic variants of the 57 P450s and their 3 redox partners was obtained as previously described (Hartz et al., 2022). Briefly, whole genome sequencing (WGS) VCF files were extracted from the Parkinson’s Progression Markers Initiative database (PPMI; July 2018 release) (Marek et al., 2018) for P450s, POR, Adx and AdR and genetic variants were identified. The identified variants were annotated with SnpEff 5.0 (Cingolani et al., 2012b) and SnpSift 5.0 (Cingolani et al., 2012a), further processed with the R programming language and classified in groups (IPD, GPD, GUN and HC) based on clinical diagnosis. Altogether 317 GPD, 344 GUN and 193 HC were included into our analyses. In addition to the genetic data, information for the intake of fish oil supplements and vitamin D by some of the patients was also obtained from the PPMI database (https://ida.loni.usc.edu/login.jsp?project=ppmi). A comparison was made, using Pearson’s chi-square test, between GUN individuals and GPD patients, for whom information in the PPMI database was available stating that they take vitamin D (or vitamin D-based medicines like Dekristol) or fish oil/omega-3 fatty acids. We aimed to assess whether the intake of these supplements would protect the genetically predisposed individuals (GUN) from getting symptoms of the disease (GPD).

IBM-SPSS Version 26 and 27 were used for statistical analysis. Due to the explorative nature of the study, we did not account for the issue of multiple statistical testing. Thus, we report raw 2-sided *p*-values without adjustment. The significance level p is set at 0.05. Risk factors are assessed using logistic regression and reported as odds ratios (OR) with 95% confidence intervals (CI) (Hartz et al., 2022). The odds ratio is a statistical measure describing the strength and direction of an association between two variables, in our case a certain SNP in a PD patient compared with HC or GUN. Calculation of OR is performed as follows (number of PD patients with a SNP/number of HC (GUN) with a SNP)/(number of PD patients without a SNP/number of HC (GUN) without a SNP). If OR is <1, the SNP is under-represented in PD patients, if it is >1, then it appears to be over-represented in PD patients compared to HC (GUN). The CI gives a measure as to how precise the OR value could be calculated.

The P450s are subdivided into 6 groups according to their main function and substrate class as suggested by Guengerich (Guengerich, 2017) and this classification is applied consistently throughout the whole study.

## Results

Our first approach was to compare the number of SNPs in GUN (individuals with genetic predisposition to PD, but without symptoms of the disease) and GPD (individuals with genetic predisposition to PD suffering from the disease) with that of the healthy individuals in the PPMI database, taken as controls (GUN/HC and GPD/HC, Table 1). Surprisingly, it turned out that both, GUN and GPD, have many SNPs, which are strongly over-represented compared to their occurrence in controls (Table 1). As can be seen from Table 1, most of the 57 P450s show SNPs with OR values >5, i.e., being more than 5 times over-represented in GUN and GPD compared to HC. Only 26 out of 57 P450s as well as the three redox partners do not show SNPs with OR values >5 when comparing GUN and HC and 31 out of 57 P450s as well as AdR and Adx when comparing GPD and HC. As shown previously (Hartz et al., 2022), this value is much higher (56 P450s and AdR) when comparing PD patients with unknown origin of the disease (IPD) and HC, where only CYP46A1 as well as Adx and POR were demonstrated to display SNPs with OR values >5 compared to HC.

**TABLE 1:** Summary of the single nucleotide polymorphisms (SNPs) with statistically significant association (*p*-value < 0.05) to Parkinson´s Disease (PD) for the 57 human cytochrome P450 genes and their 3 redox partner genes, *Adx*, *AdR* and *POR*. Compared are GPD and HC (GPD/HC), GUN and HC (GUN/HC) and GPD and GUN (GPD/GUN) and SNPs for the various comparisons are subdivided into groups based on the strength of association to PD, represented by the odds ratio (OR). SNPs with OR > 5 and OR < 0.2 are most strongly associated to PD being more than 5 times over- or underrepresented, respectively, in GPD patients when comparing GPD and HC (GPD/HC), in GUN patients when comparing GUN and HC (GUN/HC) and in GPD patients when comparing GPD and GUN patients (GPD/GUN). The cytochrome P450s (CYPs) are subdivided into 6 groups according to their main function and substrate class (Guengerich, 2017).

Substrate class	*CYP*	Total SNPs	OR < 0.2			OR = 0.2–0.5			0.5 < OR < 2.0			OR = 2.0–5.0			OR > 5		
			GPD/HC	GUN/HC	GPD/GUN	GPD/HC	GUN/HC	GPD/GUN	GPD/HC	GUN/HC	GPD/GUN	GPD/HC	GUN/HC	GPD/GUN	GPD/HC	GUN/HC	GPD/GUN
Drugs	*1A1*	86	**-**	-	-	-	-	1	-	1	-	-	-	-	-	-	-
	*1A2*	130	**-**	-	-	-	-	4	1	2	1	-	3	-	-	-	-
	*2A6*	231	6	-	-	11	2	3	2	-	2	3	4	-	2	1	-
	*2A13*	188	1	-	-	-	-	1	-	-	-	-	-	1	3	5	-
	*2B6*	584	**-**	-	-	1	-	-	69	53	4	36	56	1	1	1	-
	*2C8*	591	**-**	1	1	2	2	2	23	2	8	25	7	-	1	2	-
	*2C9*	981	**-**	-	-	1	-	-	28	1	3	3	2	2	1	2	-
	*2C18*	1935	**-**	-	-	3	4	1	5	2	4	-	1	-	1	3	-
	*2C19*	2,908	**-**	2	1	6	13	1	12	4	7	4	2	4	8	14	-
	*2D6*	153	**-**	-	-	1	2	-	3	-	3	4	2	1	3	-	-
	*2E1*	965	3	6	1	6	11	1	2	10	5	6	13	2	1	1	-
	*2F1*	336	2	1	-	1	1	4	1	4	1	2	12	-	6	6	-
	*3A4*	331	2	5	-	1	3	-	-	-	-	1	1	2	-	-	-
	*3A5*	424	1	7	-	4	2	-	-	2	-	1	-	2	-	-	-
	*3A7*	387	1	4	-	2	1	1	1	-	1	-	-	-	-	-	-
	*3A43*	548	3	34	-	6	20	-	1	3	10	3	4	1	-	2	-
Fatty Acids	*2J2*	436	-	-	-	16	20	-	9	13	-	2	2	-	-	1	-
	*2U1*	334	2	1	-	1	15	1	2	13	2	1	3	9	-	-	-
	*4A11*	238	1	1	-	2	10	-	-	1	2	9	16	-	-	-	-
	*4B1*	1,218	20	19	3	21	25	6	22	37	7	5	24	1	4	4	-
	*4F11*	547	2	9	1	39	73	-	100	23	1	-	7	2	-	2	-
	*4F12*	313	-	**1**	1	4	7	1	1	2	11	-	7	-	1	1	**-**
	*4F22*	877	1	2	-	3	3	23	16	17	4	9	35	4	-	2	-
	*4V2*	510	1	2	-	10	15	3	37	28	2	2	2	1	1	2	1
Eicosanoids	*4F2*	409	-	10	1	3	5	5	8	3	-	-	-	1	-	-	-
	*4F3*	653	4	10	-	14	5	-	20	8	37	16	**-**	5	1	-	1
	*4F8*	313	2	22	-	3	7	1	53	1	16	-	-	-	-	-	1
	*5A1*	4,394	16	9	-	77	18	12	54	41	19	48	68	6	10	3	-
	*8A1*	1,074	1	3	3	8	17	13	3	15	10	6	7	1	1	5	1
Vitamins	*2R1*	184	3	2	-	1	2	2	6	3	1	-	1	-	-	-	-
	*24A1*	424	2	2	-	6	11	-	15	25	4	20	23	5	-	3	1
	*26A1*	123	-	-	-	-	1	3	-	1	-	-	3	1	1	1	-
	*26B1*	366	-	1	-	6	2	-	6	-	5	-	-	3	1	-	1
	*26C1*	269	-	-	-	-	1	1	-	-	-	-	1	2	1	1	-
	*27B1*	79	-	-	-	-	-	-	4	-	-	-	-	-	-	-	-
	*27C1*	754	1	3	1	20	48	14	21	44	10	3	41	26	-	-	1
Sterols	*1B1*	977	7	8	-	3	11	1	2	3	2	5	5	1	-	4	-
	*7A1*	157	-	-	-	-	-	-	1	-	3	-	1	-	-	-	-
	*7B1*	2,849	5	9	-	7	11	3	1	-	6	3	8	1	7	4	5
	*8B1*	54	2	-	1	-	-	1	5	4	4	-	-	-	-	-	-
	*11A1*	413	-	1	-	-	7	-	1	12	-	8	7	1	-	2	-
	*11B1*	205	-	-	-	1	1	-	-	-	-	-	1	-	-	2	-
	*11B2*	212	-	8	-	-	1	3	1	-	1	1	1	1	-	-	-
	*17A1*	99	-	-	-	-	1	-	-	-	2	-	-	-	-	-	-
	*19A1*	2022	2	2	-	9	21	1	30	4	56	2	3	1	-	-	-
	*21A2*	102	-	1	-	7	2	-	-	1	-	7	7	-	-	-	-
	*27A1*	475	2	5	-	5	3	-	-	-	15	-	-	-	-	-	-
	*39A1*	1700	3	6	-	24	10	6	89	105	4	74	13	4	1	4	2
	*46A1*	27	1	3	-	3	2	4	1	2	15	1	5	6	3	1	-
	*51A1*	986	4	9	1	2	3	2	70	84	2	81	88	5	-	-	-
Unknown	*2A7*	246	1	-	-	4	43	-	37	39	2	-	4	-	6	6	-
	*2S1*	279	-	1	-	2	10	5	3	11	7	1	2	6	2	6	-
	*2W1*	173	1	1	-	9	32	4	5	3	9	-	-	1	-	-	-
	*4A22*	233	-	-	-	22	22	-	15	15	-	-	-	-	-	-	-
	*4X1*	385	1	1	-	31	36	-	3	9	-	1	1	-	-	-	-
	*4Z1*	781	2	8	-	119	137	-	8	7	-	2	-	-	2	6	-
	*20A1*	1,011	2	7	3	7	18	4	8	37	57	38	32	2	2	5	2
Redox Partner	*AdR*	183	-	-	-	3	12	-	1	-	-	1	1	-	-	-	-
	*Adx*	667	3	3	-	22	46	1	2	27	18	1	3	1	-	-	-
	*POR*	1797	1	10	2	23	21	2	48	28	28	17	18	4	9	-	-

Regarding P450s with SNPs displaying OR values <0.2 i.e., SNPs that are more than 5 times over-represented in HC compared to GPD or GUN, it was found that there are 23 P450s as well as AdR in GPD/HC and 18 P450s as well as AdR in GUN/HC present which do not have SNPs with OR < 0.2 (Table 1). Again, the number of P450s with OR < 0.2 is lower when comparing IPD patients with controls (37 P450s as well as AdR and Adx do not display SNPs with OR < 0.2) (Hartz et al., 2022).

To identify novel players leading to the differences between GPD and GUN individuals, i.e., to question why GUN individuals do not show symptoms of PD although they also have a genetic predisposition, while GPD individuals show such symptoms, we compared the distribution of SNPs between the two groups (GPD/GUN, Table 1; Table 2). It can be seen that the genes of 7 P450s as well as AdR do not show any significant differences between GPD patients and GUN (Table 2)**.** In contrast, three P450 genes (*CYP4V2*, *CYP24A1* and *CYP39A1*) display SNPs with OR values >10 when comparing the two groups. Most P450s fall into the group with marginal effects (OR values between 0.50 and 2.00). Differences between the two groups with OR values >5 or <0.2 are found in 10 and 12 P450s, respectively (Table 1). POR has also two SNPs that appear with OR values below 0.2 when comparing GPD and GUN individuals. Moreover, as can be seen in Table 1, considerably less SNPs are statistically different between GPD and GUN individuals (GPD/GUN) than between HC and GUN individuals (GUN/HC) or HC and GPD patients (GPD/HC). This is especially true for SNPs, which are more than five times over-represented in HC (OR < 0.2). More than 300 SNPs in 43 P450s are over-represented in HC compared with GUN and GPD patients (108 for GPD/HC and 227 for GUN/HC). In contrast, only 18 SNPs in 12 P450 genes are over-represented in GUN compared with GPD patients.

**TABLE 2:** Classification of cytochrome P450 genes (CYPs) and genes of their redox partners, Adx, AdR and POR according to the strength of association of their SNPs to Parkinson´s Disease based on the GPD/GUN comparison.

CYPs without any effect	2J2, 27B1, 11B1, 21A2, 4A22, 4X1, 4Z1, AdR
CYPs with marginal effects (OR = 0.50–2.00)	1A2, 2A6, 2B6, 2C8, 2C9, 2C18, 2C19, 2D6, 2E1, 2F1, 3A7, 3A43; 2U1, 4A11, 4B1, 4F11, 4F12, 4F22, 4V2; 4F3, 4F8, 5A1, 8A1; 2R1, 24A1, 26B1, 27C1; 1B1, 7A1, 7B1, 8B1, 11B2, 17A1, 19A1, 27A1, 39A1, 46A1, 51A1; 2A7, 2S1, 2W1, 20A1; Adx, POR
CYPs and redox partners with SNPs with ORs in the range 2.00–5.00, but no ORs >5.00	2A13, 2B6, 2C9, 2C19, 2D6, 2E1, 3A4, 3A5, 3A43; 2U1, 4B1, 4F11, 4F22; 4F2, 5A1; 26A1, 26C1; 1B1, 11A1, 11B2, 19A1, 46A1, 51A1; 2S1, 2W1, Adx, POR
CYPs and redox partners with SNPs with ORs in the range 2.00–5.00 AND ORs >5	4V2; 4F3; 8A1; 24A1, 26B1; 27C1; 7B1; 39A1; 20A1
CYPs and redox partners with SNPs with ORs >5.00	4V2; 4F3, 4F8, 8A1; 24A1, 26B1, 27C1; 7B1, 39A1; 20A1
CYPs and redox partners with SNPs with ORs <0.2	2C8; 2C19; 2E1; 4B1, 4F11, 4F12; 4F2; 8A1; 27C1; 8B1, 51A1; 20A1; POR
CYPs with OR > 5.0 and no OR <0.2	4V2; 4F3, 4F8; 24A1, 26B1; 7B1, 39A1
CYPs with OR < 0.2 and no ORs >5	2C8; 2C19, 2E1; 4B1, 4F11, 4F12; 4F2; 8B1; 51A1; POR
CYPs with ORs >10	4V2; 24A1; 39A1

**FIGURE 1:**
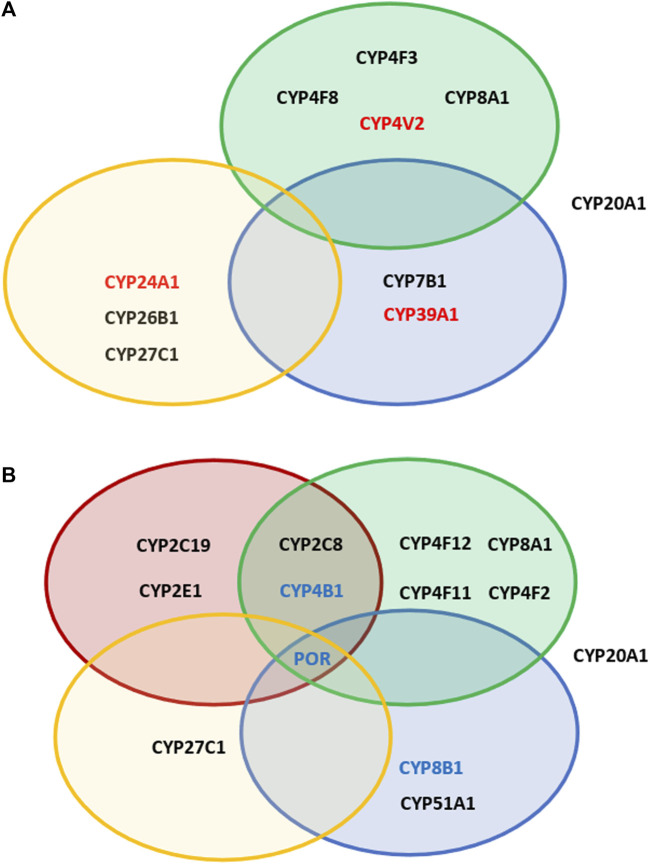
Overview of the effect of different SNPs in cytochromes P450 involved in various physiological pathways. Shown are P450s, which display SNPs with OR values >5 **(A)** and <0.2 **(B)** in GPD patients vs GUN individuals. Red circles: P450s participating in the biotransformation of xenobiotics; green circles: P450s involved in immune response and inflammation; blue circles: P450s involved in sterol metabolism or degradation of cholesterol; yellow circles: P450s involved in the metabolism of vitamins A and D. In addition, CYP20A1 is shown, which remains an „orphan“ P450, but is known to be involved in neurophysiological functions. P450s shown in red in **(A)** are those with OR values >10 (GPD/GUN). P450s shown in blue in **(B)** are those having SNPs found to be under-represented when considering both, GPD/HC and GPD/GUN.

After that, we were looking at the association of SNPs in different P450 genes concerning their over- or under-representation between GPD and GUN in the various classes of P450s, divided according to their major substrate class (Guengerich, 2017). As shown in Figure 1, SNPs with OR > 5 are found in P450 genes involved in fatty acid and eicosanoid, in vitamin as well as in sterol metabolism. Considering SNPs with OR < 0.2, besides these 3 classes also P450s involved in the biotransformation of drugs and xenobiotics are affected. In addition, in both groups (OR > 5 and OR < 0.2) CYP20A1, an orphan P450, seems to play a prominent role.

As already mentioned, there are no SNPs with OR > 5 in the drug- and xenobiotics metabolizing P450s (Figure 1; Table 1; Table 2) which show an association with the manifestation of PD (GPD/GUN). However, except for seven P450s (*CYP1A1*, *CYP1A2*, *CYP2A6*, *CYP2C8*, *CYP2C18*, *CYP2F1* and *CYP3A*), all the other enzymes in this group have SNPs which are 2-5fold over-represented in GPD compared to GUN individuals. Additionally, three P450s (*CYP2C8*, *CYP2C19* and *CYP2E1*) have one SNP each, more than five times over-represented in GUN compared to GPD (OR < 0.2) (Figure 1).

With the exception of *CYP4V2*, the fatty acid converting P450s do not seem to possess SNPs that are highly over-represented in GPD patients compared to GUN individuals (OR > 5) (Table 1; Table 2). *CYP4V2* shows one SNP that is ten times over-represented in GPD patients compared to GUN individuals (OR = 10.02). It is found in an intron region and described as a modifier of function. No SNPs with an OR value <0.2 have been found in *CYP4V2* and only one SNP has been described with an OR value between 2.0 and 5.0. Looking further into the fatty acids metabolizing group of enzymes, there are five P450 genes (out of 8) with a total of 17 SNPs with OR values between 2 and 5, and three P450 genes with 5 SNPs displaying OR < 0.2. *CYP2U1* with 9 SNPs having OR values between 2 and 5 and *CYP4B1* displaying 3 SNPs with OR < 0.2 are especially remarkable.

In contrast to the drug and xenobiotics metabolizing P450s, P450s involved in eicosanoid metabolism display significant differences between GPD and GUN. The differences can be traced to SNPs in *CYP4F3*, *CYP4F8* and *CYP8A1* showing OR values >5 for GPD/GUN. The three SNPs with OR > 5 are all located in introns and identified as modifiers of function. In addition, differences in SNPs with OR values between 2.0 and 5.0 have been found for *CYP4F2*, *CYP4F3*, *CYP5A1* and *CYP8A1* and 82 SNPs are described in the eicosanoid group with OR values between 0.5 and 2.0. Additionally, the eicosanoid group is one of the groups with the biggest number of highly protective SNPs (OR < 0.2). The described data indicates substantial variations between GUN and GPD in genes coding eicosanoid metabolizing P450s.

Some of the P450s belonging to the vitamin group also show strong differences in their SNPs between GPD and GUN (Table 1; Table 2). *CYP24A1* has one SNP with OR = 10.02 and *CYP26B1* and CYP27C1 have one SNP each with OR > 5 (OR = 6.15 and 6.25, respectively). All three SNPs are located in intron regions and described as modifiers. Additionally, *CYP27C1* has a SNP with OR < 0.2. The three P450s (*CYP24A1*, *CYP26B1* and *CYP27C1*) also have 5, 3 and 26 SNPs, respectively, with OR values between 2.0 and 5.0. Altogether, this indicates an important contribution of this group of P450 genes for the development of PD.

When considering P450s involved in sterol metabolism, it becomes obvious that mainly SNPs in those P450s are different between GPD and GUN that are also different between HC and GPD: *CYP7B1* and *CYP39A1* displaying SNPs with OR values >5 as well as *CYP46A1* with 6 SNPs showing OR values between 2.0 and 5.0. Once again, all SNPs with OR values >5 are located in intron regions and information on their exact effect on function is not available.

From the group of P450s with unknown function, *CYP20A1* plays an outstanding role. It shows two SNPs with OR > 5, two SNPs with OR values between 2.0 and 5.0 and 57 SNPs with OR values between 0.5 and 2.0. The two SNPs with OR > 5 are located in introns and described as modifiers of function.

When comparing the occurrence of special SNPs in the three groups (HC, GPD, GUN), it turns out that all SNPs with OR > 5 discussed so far are represented in GPD as well as in HC, but under-represented in GUN. This means that they show up as “protective” when compared with HC. When looking at SNPs which are either over- or under-represented in GPD/GUN and in GPD/HC as well, the picture is different. It turns out that in many cases the effect is rather moderate with OR values between 1.4 and 3.3 (Table 3). Six P450s are identified in the xenobiotics metabolizing group, 3 each in the fatty acid and in the eicosanoid group, 2 in the vitamin group, 5 in the sterol group and 1 in the group of P450s with unknown function. *CYP5A1* with 13 SNPs, *CYP2C8* with 10 SNPs, *CYP4F3* and *CYP4B1* with 7 SNPs each as well as the redox partner *POR* with 8 SNPs seem to be of special importance. While in many cases the SNPs seem to have a moderate effect on the progression of PD, some of them seem to be more important. Thus, one of the SNPs in *CYP5A1* which has an OR value of 2.13 in the GPD/GUN ratio, shows a value of 5.22 when looking at GPD and HC. This SNP is also over-represented in IPD patients compared to HC (OR = 4, data not shown). Moreover, one of the SNPs in *CYP4F3* displaying an OR value of 2.99 in the GPD/GUN ratio, shows a value of 10.21 when comparing GPD and HC. Further on, except for *CYP2D6*, *CYP3A4*, *CYP24A1* and *CYP7A1*, all 20 P450s in Table 3 as well as *POR* show SNPs with OR values <1 in GPD/GUN, some of which with OR values equal or below 0.2 (*CYP2F1*, *CYP4B1* with three such SNPs; *CYP5A1*, *CYP8B1*, *POR* with 1 SNP each). It is certainly worth mentioning that out of the 75 SNPs in all P450s and POR, 26 are over-represented in GPD patients compared to HC and GUN individuals (OR > 1), but 49 are under-represented in GPD patients compared to HC and GUN individuals, showing the important role of protective SNPs in the appearance of PD.

**TABLE 3:** Summary of all significant SNPs that are over- or under-represented in GPD patients when compared to both, HC (GPD/HC) and GUN (GPD/GUN) individuals. Marked in bold are SNPs, that are over- (OR > 1) or under-represented (OR < 1) also in IPD patients.

Substrate class	*CYP*	SNP References	GPD/GUN OR	GPD/HC OR
Drugs	*CYP2C8*	rs34115801	1.46	2.1
		rs10882520	1.44	2
		rs1341162	1.44	2
		rs1934978	1.4	2
		rs11572078	1.4	2
		rs12246157	1.4	2
		rs78705198	1.4	2
		rs1113129	1.4	2
		rs191305096	0.4	0.3
		rs117072179	0.41	0.3
	*CYP2D6*	chr22_4212700_GA	1.6	2.2
		chr22_42127207_CT	1.6	2.1
		chr22_42127941_GA	1.5	1.8
	*CYP2C9*	rs147118402	1.8	2.3
		chr10_94954906_TTTTTA	1.5	2.4
	***CYP2A6***	**rs2002976 (0/1) (IPD/HC OR = 0.6)**	**0.45**	**0.33**
	*CYP2F1*	chr19_41119717_CTCTCTCTATATATATA/*	0.2	0.22
	*CYP3A4*	rs28371763	2.6	2.9
Fatty acids	*CYP4B1*	rs201934421	3.3	3.4
		rs60103736	0.16	0.14
		rs60103736	0.24	0.21
		chr1_46800204_TTTCTCTTC	0.1	0.09
		chr1_46800203_CTTTCTTC	0.11	0.1
		chr1_46782839_CTCTT	1.4	1.6
		rs72286736	0.27	0.18
	*CYP4V2*	rs59350227	0.7	0.58
		chr4_186204405_CGGTGGAGACGTTTCGCTGGCGTAAGAGGTGGAGGTGGAGA (0/1)	0.56	0.56
	*CYP4F11*	rs3930732	0.66	0.55
Eicosanoids	*CYP5A1*	chr7_139950037_ATTTTTTATT	0.4	0.18
		chr7_139778328_TTTG	0.28	0.21
		rs143131884	0.30	0.21
		rs191028344	0.30	0.26
		rs62490128	0.2	0.11
		rs2008582	0.53	0.32
		rs78396607	0.56	0.52
		**rs2299899 (IPD/HC OR = 2.09)**	**1.67**	**3.16**
		rs10260531	0.56	0.52
		rs57244136	0.56	0.52
		rs10234650	1.39	1.79
		rs2299897	2.28	4.44
		**rs5887946 (IPD/HC OR = 4)**	**2.13**	**5.22**
	*CYP4F3*	rs4807965	2.31	2.24
		rs1915380	0.68	0.65
		rs28371492	0.68	0.65
		rs28371541	2.99	10.21
		rs139791776	0.60	0.69
		rs2733751	0.71	0.66
		rs2683041	0.71	0.65
	*CYP8A1*	rs77401275	0.32	0.33
Vitamins	*CYP26B1*	rs61138718	0.65	0.63
		rs3768647	0.62	0.63
		rs3768644	0.58	0.48
		rs10166057	0.54	0.42
	*CYP24A1*	rs6022987	1.51	1.62
		rs2762935	1.55	1.55
Sterols	*CYP39A1*	chr6_46616152_CTTTCTTTCTTTC	0.5	0.47
		chr6_46616154_TTCTTTC	0.56	0.56
		chr6_46616156_CTTTCTTTTTTCTCTTTCT	0.5	0.43
		chr6_46627420_TTA	1.45	1.57
	*CYP51A1*	rs112952169	0.61	0.52
		chr7_92128463_CGTC	0.66	0.63
	*CYP8B1*	rs6782601	0.19	0.09
		rs735320	0.24	0.18
	*CYP7A1*	rs8192879	1.61	1.51
	CYP46A1	rs76169349	0.51	0.36
Unknown	*CYP2W1*	chr7_988002_GGGGGGGTCCCCTCTGTGTGTCCT	0.42	0.38
		rs2272375	0.66	0.67
Redox partner	*POR*	rs10225188	0.51	0.65
		rs9886105	0.52	0.64
		rs10239977	0.53	0.65
		rs13231817	0.53	0.66
		rs11764251	0.53	0.67
		rs35350121	0.55	0.68
		rs116971685	0.41	0.47
		rs377474536	0.13	0.20

## Discussion

Intense worldwide investigations are carried out to identify novel markers and targets to better diagnose, understand and treat PD. There is a special lack in deciphering the detailed biochemical basis of PD to derive directions for a causative treatment of the disease. Our very recent study identified various P450 genes as novel potential players in the pathogenesis of PD (Hartz et al., 2022). A special subgroup of PD patients are those with familial PD caused by monogenetic mutations in various genes such as *LRRK2, SNCA, GBA* and others (Nalls et al., 2019; Bandres-Ciga et al., 2020).

It is well known, however, that not all individuals who have such a genetic predisposition to PD develop the disease and there are people, who, although genetically predisposed, do not show symptoms of the disease. Using the PPMI database and considering genetic differences in the 57 human P450 genes and their three redox partners, AdR, Adx and POR, we aimed to disclose the classes of P450s, the individual P450s and the specific SNPs that are important in turning genetic predisposition into symptomatic disease. We based our discussion and conclusions on two types of SNPs. First, SNPs that are over- or under-represented when comparing GPD and GUN (GPD/GUN, Table 1; Table 2) were identified regardless of whether these SNPs were also differently presented when comparing GPD/HC. Special attention was paid to strongly over- or under-represented SNPs, i.e., those with OR > 5, being more than 5 times over-represented in GPD patients compared to GUN, and those with OR < 0.2, being more than 5 times under-represented in GPD compared to GUN. The list of SNPs with OR > 5 is shown in detail (including designation of the SNP, OR values and confidence intervals) in Table 4. Second, SNPs over-represented (or under-represented) in GPD patients when compared to both, HC and GUN, i.e., which display significant OR values in both, the GPD/HC and GPD/GUN comparisons (Table 3), are considered. Possible consequences of associations between the SNPs in P450 genes and the switch from GUN to GPD are being discussed paying attention to the affected P450s and their potential metabolic impact.

**TABLE 4:** Summary of all significant single nucleotide polymorphisms (SNP) with OR > 5 when comparing GPD and GUN patients. The confidence interval (CI) of the OR is indicated by the lower and upper limit for a confidence level of 95%.

Gene	SNP	OR	95% CI		*p*-value	Comparison
			Lower	Upper		
*CYP4V2*	rs138504382^a^	10.023	1.263	79.564	0.029	GPD/GUN
					
*CYP24A1*	rs146156359^a^	10.023	1.263	79.564	0.029	GPD/GUN
					
*CYP39A1*	rs141849577^a^	10.023	1.263	79.564	0.029	GPD/GUN
	chr646616193_TCCCTCCCTC^a^	5.055	1.059	24.130	0.042	GPD/GUN
*CYP4F8*	rs189015704^a^	8.880	1.104	71.405	0.040	GPD/GUN
*CYP7B1*	rs117323804^a^	8.880	1.104	71.405	0.040	GPD/GUN
	rs117554868^a^	8.880	1.104	71.405	0.040	GPD/GUN
	rs199716748^a^	8.880	1.104	71.405	0.040	GPD/GUN
	rs149196751^a^	8.880	1.104	71.405	0.040	GPD/GUN
	rs138019891^a^	8.880	1.104	71.405	0.040	GPD/GUN
*CYP27C1*	chr2127204448_G^a^	6.249	1.370	28.510	0.018	GUN/GPD
*CYP4F3*	chr1915651701_TTATGTCT^a^	6.167	1.356	28.044	0.019	GPD/GUN
*CYP26B1*	chr272144944_GA^a^	6.147	1.352	27.952	0.019	GPD/GUN
*CYP20A1*	rs4673253^b^	5.379	1.793	16.139	0.003	GPD/GUN
	rs79425344^b^	5.379	1.793	16.139	0.003	GPD/GUN
*CYP8A1*	rs112853036^a^	5.269	1.500	18.512	0.010	GPD/GUN

^a^ Heterozygous allele combination (wild type/SNP).

^b^ Homozygous allele combination (SNP/SNP).

When analyzing the data from the PPMI database, we were first of all surprised that GPD as well as GUN individuals showed much more highly significant SNPs (OR > 5 and OR < 0.2) in their P450 genes than IPD patients when compared with healthy individuals (Hartz et al., 2022). Whether people with genetic predisposition generally have more SNPs in their genes than other individuals or whether the higher number of SNPs is directly related to the genetic predisposition is unclear and needs further investigation.

When comparing GPD and GUN, 7 P450 genes as well as AdR did not show any differences in their SNPs between GPD and GUN (Table 2), suggesting that they do not play a significant role for the progression of the disease from GUN to GPD. Ten P450 genes have SNPs with OR > 5 and 12 P450s as well as POR have SNPs with OR < 0.2. Taking a closer look at the SNPs with OR values >5 for GPD/GUN, it is interesting to note that most of those SNPs are over-represented in GPD and abundant in HC as well, but are barely found in GUN. This suggests that the SNPs are especially unfavorable when combined with a genetic predisposition (mostly mutations in *LRRK2* or *GBA* are found in these groups in the PPMI database). Interestingly, the number of these SNPs in IPD patients is also slightly (but not statistically significantly) increased compared with HC (data not shown). Taking into account that PD is a multifactorial disease and that mutations in different genes (which are not identified yet) can also be expected to contribute to the occurrence of IPD, this observation seems to be very reasonable. Interestingly, GUN individuals display a lower number of the corresponding SNP this way decreasing their disposition to the disease. The underlying mechanisms for this negative selection (in GUN, SNPs which are present in HC, GPD and even IPD, are under-represented) is not yet clear. Concerning the consequences of the SNPs with OR > 5 (Table 1), it has to be stated that all of them are found in intron regions of corresponding P450 genes and designated as modifiers of function so that differential regulation of gene expression (through binding of transcription factors, mRNAs, *etc.*) rather than a direct effect on P450s function and expression seems a plausible scenario. The finding that the SNPs are found in non-coding regions and have not been detected in previous genome-wide association studies coincides very well with observations of other laboratories (Ohnmacht et al., 2020).

When the impact of SNPs for the manifestation of GPD vs. GUN is considered concerning the class of P450 affected, it turns out that, in contrast to the GPD/HC group described previously (Hartz et al., 2022), P450s involved in the metabolism of drugs and xenobiotics, although playing some role, are not of high importance in turning GUN into GPD (Table 1). However, 6 out of 16 drug and xenobiotics metabolizing P450s show SNPs being present in GPD/HC as well as GPD/GUN. *CYP2D6*, *CYP2C9* and *CYP3A4* show OR values between 1.4 and 2.9 in both, GPD/HC and GPD/GUN, while *CYP2A6* and *CYP2F1* display protective SNPs with OR < 1. Special attention should be given to *CYP2C8*, which displays 10 SNPs, though with moderate OR values, which are found in GPD/GUN and also in GPD/HC (Table 3). Summarizing, this suggests that toxic compounds, e.g., pesticides, seem to be of rather moderate importance for the manifestation of PD in genetically predisposed individuals.

In contrast to the drug and xenobiotics metabolizing P450s, CYP4V2 belonging to the “fatty acid” family and CYP20A1 designated as an “orphan” P450 (Guengerich, 2017) as well as three P450s involved in eicosanoid, three involved in vitamin and three involved in sterol metabolism display SNPs that are highly over-represented in GPD patients compared to GUN individuals (OR > 5). This indicates that endogenous factors such as variations in the metabolism of eicosanoids, vitamins and sterols rather than exogenous factors such as variations in the degradation of external substances like xenobiotics, may play a more important role in the manifestation of PD symptoms when comparing GPD and GUN individuals.

*CYP4V2* shows a SNP with OR = 10.02 in GPD/GUN (Table 1). It also shows two protective SNPs, that are under-represented in GPD/GUN and GPD/HC (Table 3). CYP4V2 is widely expressed in different organs and tissues, also in brain (Li et al., 2004), and is described to show *ω*-hydroxylase activity on saturated and polyunsaturated fatty acids (PUFAs) of medium and long chain length (Nakano et al., 2009; Nakano et al., 2012). Mutations in CYP4V2 are the main cause for Bietti’s crystalline dystrophy (Li et al., 2004), an inherited disease of the retina. It was demonstrated that functional impairment of CYP4V2 leads to a malfunction of global lipid metabolism. Patients show a marked reduction in the transformation of fatty acid precursors into *ω*-3 PUFAs, and a decreased synthesis of eicosapentaenoic acids (20:5ω-3) as well as docosahexaenoic acids (for review see (García-García et al., 2019). Lipidomic analyses of the cells of those patients also demonstrate an accumulation of cholesterol (Hata et al., 2018). Moreover, a role of CYP4V2 in ischemic stroke due to venous thromboembolism has been shown (Yue et al., 2019; Long et al., 2022).

In view of the involvement of CYP4V2 in the metabolism of saturated and polyunsaturated fatty acids, we checked whether the consumption of fish oil and omega fatty acid supplements has an effect on the manifestation of GPD since for some individuals in the PPMI database corresponding information is available. Analysis of the data, however, did not show any statistically significant differences in the number of GUN and GPD in dependence on the intake of fish (or krill) oil (21.2% (73 out of 344) GUN vs. 15.5% (49 out of 317) GPD, *p* = 0.056), thus demonstrating no protective role of the oil on the manifestation of the disease. However, it cannot be excluded that a sub-group of individuals, e.g., people showing defects in the biosynthesis of anti-inflammatory compounds derived from fatty acids, will profit from the intake of fish or krill oil.

Analyzing the role of P450s of the “eicosanoid” group, our results show that P450s involved in eicosanoid metabolism and inflammation seem to have a key function in the manifestation of GPD. Indeed, 3 out of 5 P450s involved in eicosanoid metabolism also have SNPs with OR values >5 (*CYP4F3*, *CYP4F8* and *CYP8A1*) suggesting an important effect of eicosanoids in differentiating between GPD and GUN. A similar importance of eicosanoids was described by (Hartz et al., 2022) for the association between SNPs in these genes and PD. In addition, 4 P450s (*CYP4F2*, *CYP4F3*, *CYP5A1*, *CYP8A1*) of this group have SNPs with OR values between 2 and 5 (Table 1). Moreover, *CYP5A1* displays 13 SNPs when considering simultaneously GPD/GUN and GPD/HC with OR values up to 5.2 in GPD/HC and >2 in GPD/GUN (Table 3). CYP5A1 is a thromboxane synthase and its function is related to the action of CYP8A1, a prostacyclin synthase. While both P450s use prostaglandin H_2_ (PGH_2_) as a substrate, prostacyclin (prostaglandin I_2_, PGI_2_), resulting from the catalytic action of CYP8A1, is a potent vasodilator and platelet antiaggregatory eicosanoid, while thromboxane A_2_ (TXA_2_), resulting from the catalytic action of CYP5A1, is a potent inducer of platelet aggregation and vasoconstriction. The balance of the two products affects important physiological processes such as blood pressure regulation, blood coagulation and inflammation (Rendic and Peter Guengerich, 2018). PGI_2_ is neuroprotective in neuronal cultures and in the ischemic brain with pronounced effect on microglia (Lin et al., 2002; Tsai et al., 2005). Moreover, its enhanced synthesis decreases glial activation and alleviates motor impairments in hemiparkinsonian rats (Tsai et al., 2013). Increased TXA_2_, in turn, has been demonstrated to be involved in amyloid beta protein (Abeta)-induced dysfunction of the dopaminergic nigrostriatal pathway and motor function deficits in Abeta-injected rats (Yagami et al., 2004). In addition, CYP4B1 and CYP4F3, also involved in fatty acid and eicosanoid metabolism, show differences in the occurrence of SNPs when comparing GPD/GUN as well as GPD/HC (Table 3). *CYP4B1* has 7 SNPs, one of them with OR > 3 in both cases. *CYP4F3* has also 7 such SNPs, one of them with OR = 10.21 for GPD/HC and OR = 2.99 for GPD/GUN. Interestingly, *CYP2C8* with 10 SNPs, besides playing an important role in drug and xenobiotics metabolism, possesses also epoxygenase activity and metabolizes long-chain polyunsaturated fatty acids such as arachidonic acid, eicosapentaenoic acid, docosahexaenoic acid, and linoleic acid to epoxide products (Rifkind et al., 1995). Taken together, this data underlines the remarkable importance of inflammation on the manifestation of the disease based on a genetic predisposition to PD.

When analyzing P450s involved in the metabolism of vitamins concerning differences in SNPs between GPD and GUN, it becomes obvious that again three P450s play an important role (Table 1; Table 4). *CYP24A1* is one of the three P450s displaying a SNP with OR = 10.02 for GPD/GUN. The SNP is located in an intron region and suggested to behave as a modifier of function. It has also two SNPs that are over-represented in GPD patients compared to HC and GUN with OR values in the range 1.5–1.6 (Table 3). CYP24A1 is responsible for the degradation of 25OH- as well as 1α, 25-dihydroxy vitamin D3 to 24-hydroxylated products (Chen et al., 1993; Sakaki et al., 2000). It is a central player in vitamin D homeostasis and in this way in vitamin D function. Since the precursor of vitamin D3, 7-dehydrocholesterol, is also the precursor of cholesterol, the biosynthesis of vitamin D is closely related to sterol metabolism and steroid hormone biosynthesis (Schuster, 2011; Saponaro et al., 2020). Changes in the expression and functionality of CYP24A1 may thus have broad consequences. First, malfunction could lead to an accumulation of the precursor 7-dehydrocholesterol and in this way to increased levels of cholesterol. On the other hand, an unbalanced high expression of CYP24A1 may lead to vitamin D deficiency thereby causing various disorders connected to bone metabolism, immunomodulation and proliferative disorders like cancer (Schuster et al., 2006). Very recently it was found that vitamin D activation in astrocytes could play an important role in PD pathogenesis and a protective role of vitamin D was suggested (Mazzetti et al., 2022). Moreover, a pathogenic role for CYP24A1 in multiple sclerosis and ischemic stroke has been proposed. It was shown that the risk allele rs2248359_C, which is associated with an increased expression of CYP24A1 in frontal cortex, increases the risk of multiple sclerosis, possibly by changing immunomodulation in brain (Ramasamy et al., 2014). CYP24A1 genetic polymorphisms were also shown to be significantly associated with the occurrence of an ischemic stroke (Yang et al., 2020). In this relation, it should be mentioned that overexpression of CYP24A1 could serve as a possible drug target as successful attempts for selective inhibition of CYP24A1 have already been made (Schuster et al., 2006; Alshabrawy et al., 2022). Due to the importance of CYP24A1 in vitamin D metabolism, we checked whether there is a difference in the manifestation of the disease in dependence on the intake of vitamin D as a supplement, since data for some individuals are available in the PPMI database. However, we found no statistically significant difference in the total number of GPD vs. GUN taking vitamin D (39.5% (136 out of 344) GUN vs. 33.1% (105 out of 317) GPD, *p* = 0.087) suggesting that there is no effect of nutritional supplementation with vitamin D on the manifestation of PD in genetically pre-disposed individuals. Besides *CYP24A1*, also *CYP27C1* and *CYP26B1* from the vitamin group display SNPs with OR > 5 in GPD patients compared with GUN individuals (Table 1). CYP27C1 so far has not been extensively studied. It is known that it does not catalyze the oxidation of vitamin D_3_, 1α- or 25-hydroxy vitamin D_3_ or cholesterol at detectable levels as do its relatives CYP27A1 and CYP27B1 (Kakimoto et al., 2022), but it was shown to catalyze the 3,4-desaturation of retinoids (Wu et al., 2006; Kramlinger et al., 2016). CYP27C1 is highly expressed in skin (Johnson et al., 2017), but was also shown to be expressed in the brain (https://www.proteinatlas.org). More information is available on CYP26B1. Human CYP26B1 was first described in 1999 (Nelson, 1999) and shown to be responsible for the generation of several hydroxylated forms of retinoic acid in the human brain thus playing an important role in protecting it from exposure to retinoids (White et al., 2000). Retinoic acid, which is synthesized from vitamin A, is an essential regulator of gene expression and its homeostasis is of great importance for a balanced metabolism and for neuronal differentiation. In human neurons, this homeostasis is maintained by anabolic as well as catabolic enzymes such as the catabolic P450s CYP26A1 and CYP26B1 (Stoney et al., 2016). Knock-out of the two enzymes in mice is embryonic lethal while postnatal global deletion results in severe dermatitis, blepharitis, splenomegaly, lymphadenomegaly, systemic inflammation and reduced life span (Snyder et al., 2020). The authors further concluded that CYP26B1, but not CYP26A1, is the main post-natal enzyme clearing all-*trans*-retinoic acid, which coincides well with our results showing that many SNPs in CYP26B1 (but not CYP26A1) display differences (under- or over-representation) in GPD vs. GUN and thus in the manifestation of PD.

Sterol-converting P450s also show a strong association of SNPs in corresponding P450 genes with the manifestation of PD in GPD vs. GUN. *CYP39A1* is the third P450 found in our studies with an OR = 10.02 (GPD/GUN). Moreover, it possesses one SNP with OR = 1.5 for GPD/GUN and 1.6 for GPD/HC. This P450 plays a prominent role in the degradation of cholesterol. It converts 24S-hydroxycholesterol, formed by CYP46A1 from cholesterol, into 7α,24S-dihydroxycholesterol (Li-Hawkins et al., 2000). Further hydroxylation by CYP8B1 and CYP27A1 leads to formation of bile acids. CYP39A1 is mainly localized in liver, but also found in the brain and in the eye (Baloni et al., 2020; Li et al., 2021). It was shown to be directly involved in neural cholesterol clearance and changed bile acid metabolism in Alzheimer’s disease cohorts (Baloni et al., 2020). Increasing the expression of CYP39A1 could be beneficial to reduce brain 24S-hydroxycholesterol, whose accumulation has been shown to be related to oxidative stress and reported in the brain of patients with dementia and Alzheimer’s disease (Matsuoka et al., 2020). Interestingly, using both metabolomic as well as epigenetic approaches, bile acid metabolism, being closely related to cholesterol degradation, was demonstrated to be the top perturbed metabolic pathway in the brain of PD patients (Vishweswaraiah et al., 2022) underlining the role of cholesterol degradation for the manifestation of PD as described here. Finally, using GWAS investigations it was reported that four intron variants of *CYP39A1* (rs6907129, rs6905960, rs7749491, rs16874881) are strongly associated with the occurrence of levodopa-induced dyskinesia in patients aged over 50 years at the onset of PD (Ryu et al., 2020). *CYP7B1* is another P450 gene having SNPs with OR values >5 in GPD/GUN. It shows five such SNPs, all of which with the same OR value of 8.88 and all of which clustered in the same patients. CYP7B1 is also involved in the degradation of cholesterol leading to 7α-hydroxylation of 27-hydroxycholesterol, which is formed from cholesterol with the participation of CYP27A1. The latter one is found ubiquitously while CYP7B1 mRNA transcripts are mostly found in tissues involved in steroid and bile acid biosynthesis (brain, testes, ovaries, prostate, liver) as well as reabsorption (colon, kidney, and small intestines) (Wu et al., 1999). It has been shown that the blood-brain barrier is permeable to hydroxylated cholesterol metabolites and that 24-hydroxycholesterol, which is the substrate of CYP39A1, is formed almost exclusively in the brain, while 27-hydroxycholesterol, the substrate of CYP7B1, is mainly supplied to the brain (Björkhem et al., 2018). Interestingly, Björkhem and coworkers found that the level of oxysterols in the cerebrospinal fluid may reflect neurodegenerative changes occurring in PD patients (Björkhem et al., 2013), which is in line with the association between SNPs in *CYP39A1* and *CYP7B1* and the manifestation of PD symptoms found in our study. Moreover, defects in *CYP7B1* have been associated with hereditary spastic paraplegia type 5, a group of neurodegenerative disorders characterized by progressive neurodegeneration of the corticospinal tract motor neurons (Schöls et al., 2017; Chou et al., 2020; Prestsæter et al., 2020). Hence, defects in CYP7B1 might make an individual more vulnerable for neurodegeneration in general, i.e., also for disorders besides PD.

Finally, CYP20A1, belonging to the group of “orphan” P450s, displays SNPs with OR > 5 (GPD/GUN). It has two SNPs with OR = 5.38 that are both found in the same patients. CYP20A1 is especially abundant in *S. nigra* and the hippocampus of human brain (Stark et al., 2008) and was very recently shown to have important neurophysiological functions in zebra fish (Lemaire et al., 2016; Brun et al., 2021). Unfortunately, so far only aniline and some luminogenic substances have been shown to be weakly active with CYP20A1 expressed in yeast (Durairaj et al., 2019) indicating on possible endogenic substrates (Brun et al., 2021).

Interestingly, *POR*, which did not show SNPs with OR > 5 when comparing all SNPs between GPD and GUN, displayed 4 SNPs with OR values between 2 and 5 (Table 1) as well as 8 SNPs with OR values <1 when analysing SNPs in GPD/HC and GPD/GUN (Table 3). This points to a possible modulatory effect of this redox partner on the manifestation of PD in GPD patients.

Taken together, we were able, to the best of our knowledge for the first time, to identify SNPs in the genes of proteins, which are associated with the switch from GUN individuals (with a predisposition to PD, but without symptoms of the disease) to GPD (with disease symptoms). Most impressive, some of the SNPs are up to 10 fold over-represented in GPD patients vs. GUN individuals indicating a significant role of these SNPs for the manifestation of the disease. Out of the 57 human P450s and their three redox partners (Adx, AdR and POR), 10 P450s show SNPs with OR values >5 when comparing GPD and GUN. Since those SNPs occur under-represented in GUN compared with GPD and even HC, it seems that they are not sufficient to cause PD, when occurring alone. They seem to exert their effect only when combined with other SNPs, e.g., of the predisposition genes as found here, or with SNPs in other so far unidentified genes as proposed for IPD patients. This defines important novel players in the pathogenesis of PD, especially when looking at patients with a genetic predisposition. Altogether, this underlines the well-accepted multivariant basis for the development of PD. Limitations of the study are the lack of an analysis of potential combinations of various SNPs in the different P450s of individual GPD patients and GUN individuals as well as the lack of experimental studies. However, those were outside the focus of this paper and common to most of the communications on identified PD risk factors reported so far. Comparison of the age of GPD patients at the time of PD diagnosis with GUN individuals at the time when listed in the PPMI database, shows no statistically significant difference between the two groups (58.2 ± 1.6 (GPD) vs. 57.6 ± 0.7 (GUN) years, *p* = 0.78). Nevertheless, it cannot be excluded that GUN individuals might develop symptoms of the disease at a later stage. However, taking into account that a genetic predisposition is mostly related to an early onset of the disease (Ylikotila et al., 2015), we believe that the average age of 57.61 years for GUN (at which they were enrolled in the PPMI study), is already the age at which the first PD symptoms, if any, would have developed. Interestingly, the P450s with the greatest effect on the switch from GUN to GPD do not belong to the group involved in drug and xenobiotics metabolism, like in the case of GPD/HC, but to fatty acid/eicosanoid, vitamin and sterol metabolism (Figure 1), which is further confirmed when examining also the SNPs occurring with similar OR values in GPD patients compared to both, HC and GUN individuals. Thus, endogenous rather than exogenous factors seem to be responsible for the differentiation between GPD and GUN and inflammation, vitamin A and D metabolism, cholesterol degradation and pathways affected by CYP20A1 seem to be key players in eliciting PD manifestation. Additional to deleterious SNPs that are highly over-represented in GPD patients, protective SNPs are equally important. These SNPs are largely missing in GPD but are present in GUN (Figure 1). Twelve P450s have SNPs with OR values below 0.2 when comparing GPD and GUN individuals and two P450s have SNPs with OR values below 0.2 when comparing GPD both with GUN and HC. The *CYPs* with protective SNPs belong to all substrate classes. Moreover, also *POR* shows SNPs with OR < 0.2 in both approaches indicating on a contribution of this protein, which is the redox partner of all microsomal P450s, for the transformation from GUN to GPD. Further studies, especially experimental investigations are needed to examine the consequences of the SNPs found in this study, characterize in detail biochemical pathways being affected and identify possible novel potential targets for PD early diagnostics, intervention and treatment.

## Data Availability

The original contributions presented in the study are included in the article, further inquiries can be directed to the corresponding authors.

## Abbreviations

CYP, cytochrome P450; P450, cytochrome P450; Adx, adrenodoxin; POR, NADPH-dependent cytochrome P450 reductase; *CYP*, *POR*, *Adx*, *AdR* designate the corresponding genes; PD, Parkinson’s disease; IPD, idiopathic (sporadic) PD; GPD, PD with a genetic predisposition; GUN, persons with genetic predisposition for PD, but without symptoms of disease; PPMI, The Parkinson’s Progression Markers Initiative; OR, odds ratio; CI, confidence interval.
